# A Rare Heterozygous MYH11 Missense Variant in a Patient With Recurrent Strokes, Intracerebral Arterial Pathology, and Aortic Aneurysm

**DOI:** 10.7759/cureus.91418

**Published:** 2025-09-01

**Authors:** Haley E Huggins, Oleksandr Olifir, Rodica E Petrea, Cory Siegel, Natasha Y Frank, Viken L Babikian

**Affiliations:** 1 Neurology Department, Boston University Medical Center, Boston, USA; 2 Genetics Division, Brigham and Women's Hospital, Boston, USA; 3 Neurology Department, VA Boston Healthcare System, Boston, USA; 4 Neuroradiology Department, VA Boston Healthcare System, Boston, USA

**Keywords:** case report, cerebral arteriopathy, cerebrovascular disease, myh11 variant, stroke

## Abstract

We present a patient with complex cerebral and arterial pathology found to have a rare heterozygous myosin heavy chain 11 (*MYH11)* missense variant. A 58-year-old male was seen after recurrent strokes at ages 43, 52, and 55. Brain magnetic resonance angiography (MRA) showed five outpouchings or small aneurysms arising from the intracranial internal carotid arteries (ICAs), a larger basilar artery aneurysm, and severely stenotic segments of several intracranial arteries. Brain magnetic resonance imaging (MRI) showed a mixture of small and large artery old infarcts and white matter disease. The descending thoracic aorta and the abdominal aorta were both aneurysmal on computerized tomography angiography. Whole exome sequencing (WES) identified a rare heterozygous *MYH11*: c.3818G>T, p.(Arg1273Leu) missense variant. This case report highlights the risk for vascular abnormalities, including cerebrovascular disease, in *MYH11 *pathogenic variants and underscores the importance of vigilant monitoring and early prophylactic interventions for stroke prevention in this patient population.

## Introduction

Several genetic disorders are associated with cerebral arterial disease [1-4]. In Marfan's Syndrome, fibrillin-1 defect impacts the integrity of the extracellular matrix, increasing the risk of arterial dissection [5]. In Ehlers-Danlos Syndrome, the production of collagen is disrupted, which can lead to dissection of large cerebral arteries [5]. Disease-causing variants in the neurogenic locus notch homolog protein 3 (*NOTCH3*) gene lead to Cerebral Autosomal Dominant Arteriopathy with Subcortical Infarcts and Leukoencephalopathy (CADASIL) Syndrome [6], and pathogenic variants in the high-temperature requirement factor A1 (*HTRA1*) gene are associated with cerebral microbleeds; both disorders of small brain arteries [7].

The actin alpha 2 (*ACTA2*) gene encodes a smooth muscle contractile protein, and it is relatively well studied [8-11]. Pathogenic variants in this gene are associated with diffuse smooth muscle dysfunction and various vasculopathies, including ischemic cerebrovascular disease, thoracic aortic aneurysm and dissection (TAAD), persistent ductus arteriosus (PDA), and more [8]. *ACTA2 *cerebral arteriopathy has a distinct angiographic appearance characterized by dilation of the proximal carotid arteries, stenosis, straightening, and "broomstick-like" intracranial arteries, and absence of basal lenticulostriate collaterals [9-11]. 

Similar to the *ACTA2 *gene, the *MYH11 *gene encodes another contractile protein in smooth muscle, the myosin heavy chain, critical for maintaining vascular wall stability [12]. Previous investigators have reported patients with a cerebral arteriopathy, often associated with stroke in children and young adults [12-19], but available clinical data remain very limited. Less than 12 patients with cerebral arteriopathy and *MYH11 *variants were identified in a PubMed search in June 2025. These cases ranged in age from the newborn to young adults and described varying cerebral arteriopathies, including aneurysms, arterial stenosis, occlusion, and straightening, as well as moyamoya changes [4, 12-16, 18]. Cerebral infarction and hemorrhage were reported, though not present in all cases.

Here we describe a patient, with recurrent ischemic strokes manifested in the fourth and fifth decades of life, multiple intracranial arterial aneurysms and stenotic lesions, and aortic aneurysms, who was found to be a heterozygous carrier of a rare missense variant in the *MYH11 *gene.

## Case presentation

A 58-year-old male with a history of hypertension, hyperlipidemia, chronic kidney disease, and coronary artery disease developed ischemic strokes at ages 43, 52, and 55. Although available clinical information is limited, it is suspected that the vascular risk factors were not adequately treated, particularly between the ages of 53 and 57, when the patient lived outside the United States and did not have easy access to medical care. After his third clinical stroke, he had persistent mixed transcortical aphasia, visual field deficits, and right-sided hemiparesis. He developed memory problems as well as inattention and impulsivity. By age 57, he was completely dependent on help with all activities of daily living and was diagnosed with vascular dementia. The patient is adopted; therefore, family history was limited. However, through direct-to-consumer ancestry DNA testing, he found that his biological mother had multiple strokes. 

**Figure 1:**
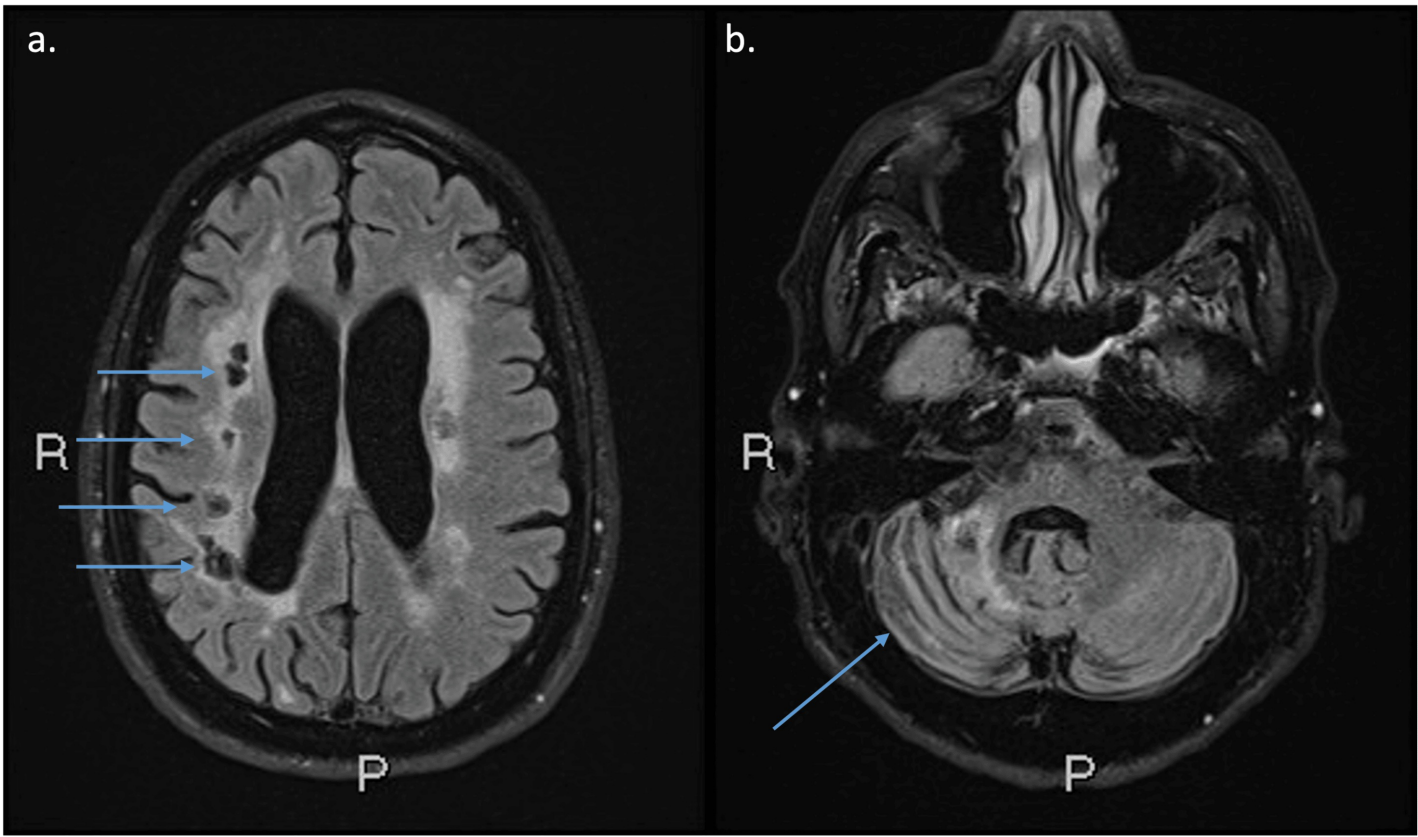
Axial T2 FLAIR fat-saturated MRI images. Axial T2 fluid attenuated inversion recovery (FLAIR) fat-saturated MRI images demonstrate multiple chronic infarcts (arrows) in the right coronal radiata (a) and a chronic infarct in the right cerebellum (b). The patient also had a focus of infarct in the left corona radiata and right thalamus (not pictured). The patient demonstrated mixed transcortical aphasia, which can likely be explained by involvement of the left arcuate fasciculus.

**Figure 2:**
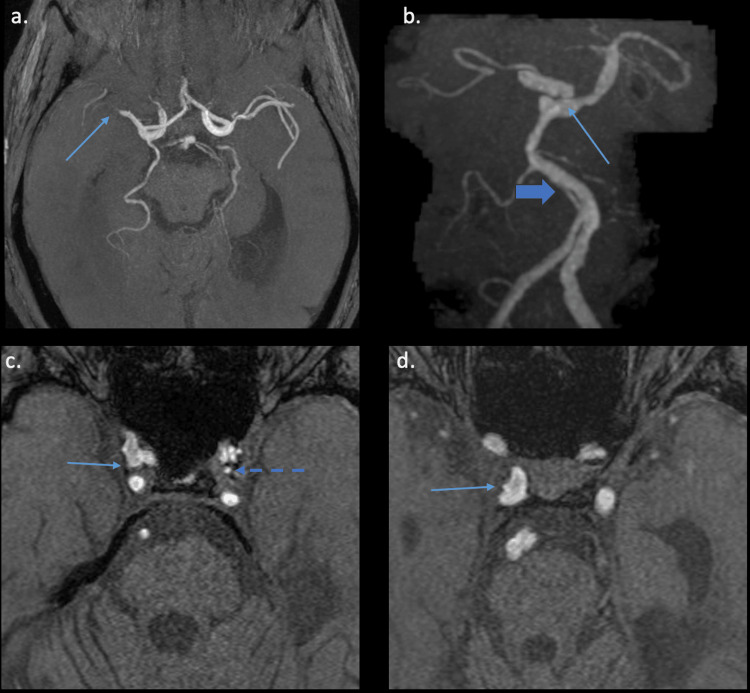
3D TOF MRA images (a, c, d) with coronal 3D MIP reconstruction (b) showing intracranial stenosis and multiple intracranial aneurysms. 3D Time of Flight (TOF) magnetic resonance angiography (MRA) images (a, c, d) with coronal 3D maximum intensity projection (MIP) reconstruction (b) showing intracranial stenosis and multiple intracranial aneurysms - (a) critical stenosis of the M1 segment of the right middle cerebral artery (arrow); (b) an 8 mm bilobed aneurysm of the distal basilar artery (thin arrow). Proximal basilar artery fenestration was also incidentally present (thick arrow); (c) 3 mm right distal cavernous internal carotid artery (ICA) aneurysm (arrow) and a 2 mm left distal cavernous ICA aneurysm (dashed arrow). D: 2 mm aneurysm or infundibulum at the lateral cavernous segment of the right ICA at the inferolateral trunk origin (arrow).

A brain magnetic resonance imaging (MRI) obtained at age 57 showed a mixture of small and large artery chronic infarcts and white matter disease (Figure 1). Multifocal chronic infarcts were seen in the right greater than left corona radiata, right cerebellum, and right thalamus. Brain magnetic resonance angiography (MRA) at age 55 showed five outpouchings or small aneurysms arising from the intracranial internal carotid arteries (ICAs), a 7.6x4.6 mm aneurysm of the basilar artery, and severely stenotic lesions of the intracranial ICAs, middle cerebral arteries, and posterior cerebral arteries bilaterally, as well as the basilar artery (Figure 2). Chest computed tomography showed that the distal thoracic aorta and the abdominal aorta were both aneurysmal.

The stroke workup included negative sedimentation rate, syphilis, and human immunodeficiency virus blood tests. The transthoracic echocardiogram showed left ventricular hypertrophy, bi-atrial dilatation, and proximal ascending aorta dilatation at 3.9 cm. A 4-day cardiac rhythm monitor did not detect atrial fibrillation.

During the past 2 years, the patient has been maintained on dual antiplatelet therapy with aspirin and clopidogrel for secondary stroke prevention. Hypertension and hyperlipidemia were addressed, and their control varied and generally improved. A revascularization surgery was not considered, given the type of intracranial cerebral arterial pathology, and there was no hemodynamically significant extracranial internal carotid artery stenosis.

Genetic testing was initially performed in November 2023 using the clinical Invitae Hereditary Cerebral Small Vessel Disease Panel, which included amyloid precursor protein (*APP*), cysthionine beta-synthase (*CBS*), collagen type IV (*COL4A1 *and *COL4A2*), cystatin C (*CST3*), Forkhead box C1 (*FOXC1*), galactosidase alpha (*GLA*), *HTRA1*, *NOTCH3*, and three prime repair exonuclease 1 (*TREX1*) genes with no reportable variants identified.

Subsequently, the clinical whole exome sequencing WES performed by the GeneDx® laboratory (GeneDx, Boston, USA) in February 2024 revealed a heterozygous variant of uncertain significance (VUS), c.3818G>T, p.(Arg1273Leu), in the *MYH11 *gene. This variant is classified as a VUS based on American College of Medical Genetics and Genomics (ACMGG) guidelines for variant interpretation [20]. In silico, this variant was predicted as damaging by MutationTaster (score 0.999), SIFT (score 0.971), and Provean (score -5.43) and as probably damaging by PolyPhen (score 0.994) algorithms. The variant was not observed in large population cohorts according to the Genome Aggregation Database (gnomAD). Based on the ACMGG criteria PM2 and PP3, the variant was reported as a VUS. This variant was also reported as a VUS to ClinVar in 2023 by Ambry Genetics. The variant segregation data within the family are unavailable.

## Discussion

We report a case of a rare heterozygous *MYH11*:c.3818G>T, p.(Arg1273Leu) missense variant in a patient with multiple brain infarcts early in life, multiple intracranial arterial aneurysms and stenotic lesions, aortic aneurysmal formation, and a familial history of stroke. 

Histologic analyses of aortas from individuals with *MYH11*-associated familial TAAD reveal smooth muscle cell disarray and focal hyperplasia in the aortic media, leading to significant narrowing in some vessels [13]. *MYH11 *is specific to smooth muscle cells and can broadly affect arterial integrity. Pathogenic *MYH11 *variants are an established cause of familial TAAD, often associated with PDA [19]. Although TAAD [12], PDA [12], and renal arterial disease [18] have often been detected in patients with *MYH11 *variants and cerebral arteriopathy, non-cerebral manifestations are relatively rare. The cerebral arterial pathology was quite variable and included aneurysms [15, 16], moyamoya changes [4, 18], and arterial stenosis, occlusion, and straightening [4, 12-14]; the latter is similar to the *ACTA2 *“broomstick arteriopathy”. Our patient is remarkable in that the arterial pathology includes several aneurysms, arterial stenosis, and basilar artery fenestration. The concurrent aortic aneurysms in this case provide further support to the notion of a disorder of arterial wall structure.

Phenotypic variability and reduced penetrance have previously been noted [17, 19], including in a report of monozygotic twins with a *MYH11* variant [19], and it has been suggested that additional genetic factors may contribute to the gene expression either by themselves or by interacting with the *MYH11 *variant. We propose that this oligogenic model [17] and other vascular risk factors could affect the onset, phenotype, and progression of the disease [17].

The *MYH11 *gene contains 41 exons and is located on chromosome 16p13.13-p13.12. Pathogenic variants in both splice sites and exons may lead to reduced smooth muscle contractility and structural support in arterial walls, predisposing to arterial wall dilation, thickening, and luminal stenosis [12-14]. The majority of TAAD-related variants reported in the *MYH11 *gene are missense changes and follow an autosomal-dominant pattern of inheritance [19]. So far, pathogenic variants have been reported predominantly in the functional domains of the myosin head and tail [13]. 

**Figure 3:**
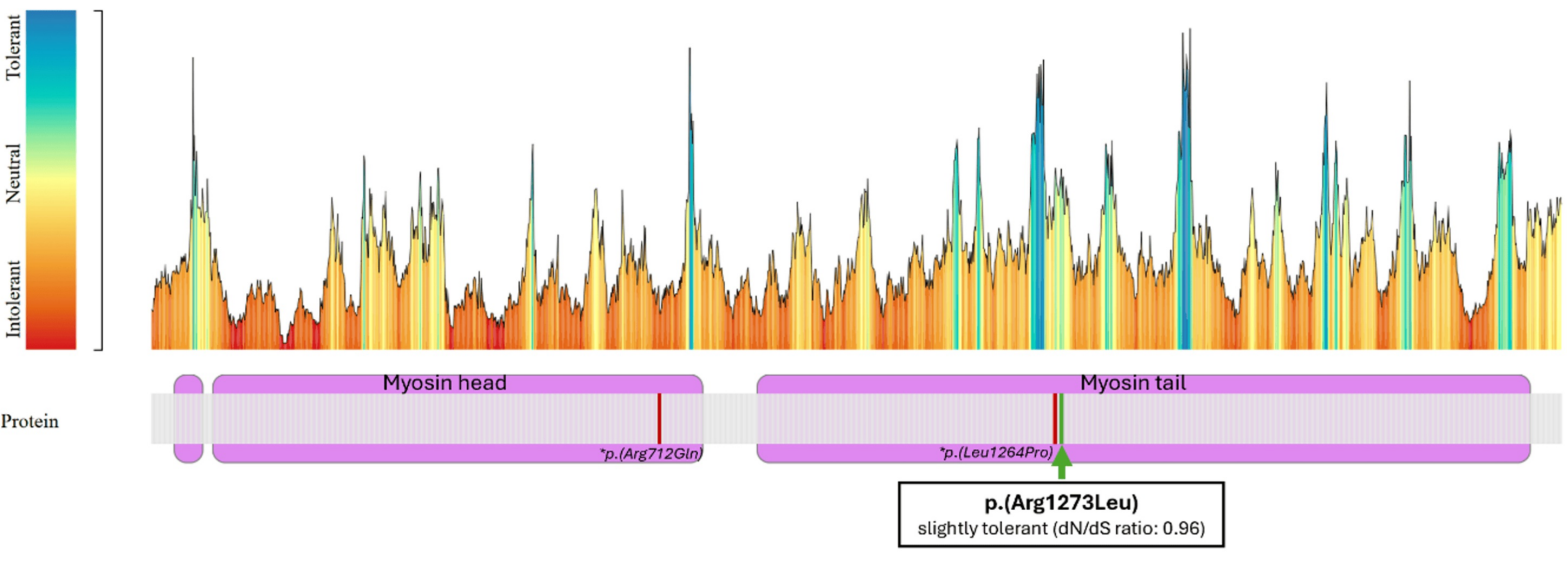
The tolerance landscape plot of MYH11 (NM_02474.2) generated using the MetaDome web server. The position of the *MYH11*:c.3818G>T, p.(Arg1273Leu) variant is marked by a green arrow. The topography of the pathogenic variants associated with familial thoracic aortic aneurysm and dissection (TAAD) reported in ClinVar is depicted by red lines. MetaDome by Radboud University Medical Center, Nijmegen, the Netherlands.

The *MYH11 *gene is intolerant to missense variants, based on a Z score of 3.43, according to gnomAD, suggesting that any damaging missense variant may significantly disrupt protein function. The reported c.3818G>T, p.(Arg1273Leu) is located in the myosin tail domain in proximity to a published variant c.3791T>C p.(Leu1264Pro) associated with familial thoracic aortic aneurysm and aortic dissection [8]. While this region is highly constrained based on the overall dN/dS ratio of < 1, the dN/dS ratio for the c.3791T>C p.(Leu1264Pro) variant of 0.96 indicates its mild tolerability (Figure 3). Thus, despite the potential association with the patient's phenotype, evidence regarding the clinical significance of this variant is still uncertain, and additional functional studies are needed for an improved understanding of its pathogenicity. 

## Conclusions

We report a case of a rare heterozygous *MYH11*:c.3818G>T, p.(Arg1273Leu) missense variant in a patient with severe cerebral arterial and aortic pathology and clinical and radiological brain infarcts. 

Young patients with unusually severe small and large arterial cerebrovascular disease and aortic aneurysms may suffer from *MYH11* or other gene pathogenic variants, and a high degree of suspicion by their clinicians will prompt appropriate testing. Additionally, this case suggests that patients diagnosed with *MYH11 *variants should receive routine cerebrovascular monitoring. Early detection of an intracranial aneurysm can allow for elective surgical treatment before rupture. Progressive carotid or basilar artery stenosis may prompt consideration for medical or surgical therapy, including treatment for common vascular risk factors for stroke. A combination of vigilant monitoring, prophylactic interventions, and risk factor management is necessary for stroke prevention in *MYH11 *variant carriers. Additionally, at-risk relatives of such patients should consider similar vascular monitoring and genetic testing given the gene’s autosomal dominant pattern of inheritance.
